# A Computational Method for Prediction of Excretory Proteins and Application to Identification of Gastric Cancer Markers in Urine

**DOI:** 10.1371/journal.pone.0016875

**Published:** 2011-02-18

**Authors:** Celine S. Hong, Juan Cui, Zhaohui Ni, Yingying Su, David Puett, Fan Li, Ying Xu

**Affiliations:** 1 Department of Biochemistry and Molecular Biology, and Institute of Bioinformatics, University of Georgia, Athens, Georgia, United States of America; 2 Department of Pathogenobiology, Jilin University, Changchun, Jilin, China; 3 College of Computer Science and Technology, Jilin University, Changchun, Jilin, China; Dana-Farber Cancer Institute, United States of America

## Abstract

A novel computational method for prediction of proteins excreted into urine is presented. The method is based on the identification of a list of distinguishing features between proteins found in the urine of healthy people and proteins deemed not to be urine excretory. These features are used to train a classifier to distinguish the two classes of proteins. When used in conjunction with information of which proteins are differentially expressed in diseased tissues of a specific type *versus* control tissues, this method can be used to predict potential urine markers for the disease. Here we report the detailed algorithm of this method and an application to identification of urine markers for gastric cancer. The performance of the trained classifier on 163 proteins was experimentally validated using antibody arrays, achieving >80% true positive rate. By applying the classifier on differentially expressed genes in gastric cancer *vs* normal gastric tissues, it was found that endothelial lipase (EL) was substantially suppressed in the urine samples of 21 gastric cancer patients *versus* 21 healthy individuals. Overall, we have demonstrated that our predictor for urine excretory proteins is highly effective and could potentially serve as a powerful tool in searches for disease biomarkers in urine in general.

## Introduction

The rapid advancement of *omic* techniques in recent years has made it possible to search for biomarkers for specific human diseases in a systematic and comprehensive manner, which is substantially improving our ability to detect diseases at early stages. Most of the previous biomarker studies have been focused on serum markers [1], mainly because of the known richness of serum in containing signals for various physiological and pathophysiological conditions.

Compared to serum markers, existing urinary markers are mostly related to urinary-tract or closely associated diseases. Only within the last few years has improved proteomic analyses of urine samples revealed that, like sera, urine is also a rich source of information for detecting human diseases such as the graft-*versus*-host disease and coronary artery disease [2], [3], [4]. Note that urine is formed by filtration of blood through the kidneys; hence some proteins in blood may pass through the filters and be excreted into urine. As a result, the urinary proteins not only reflect the conditions of the kidney and the urogenital tract, but also those of other organs that may be distal from the kidney, as at least 30% of the urinary proteins are not originally from the urogenital tract [5], [6]. The plethora of information in urine makes it an attractive source for biomarker screening since, compared to serum, the composition of urine is relatively simple, and urine collection is easier and noninvasive [7], [8].

Marker identification in urine could potentially be done through comparative proteomic analyses of urine samples of patients with a specific disease and control groups. The challenge in such searches for urinary markers in a blind fashion is twofold. (a) Urine could have a large number of proteins/peptides (in contrast to the previous understanding [8]) with relatively low abundance. (b) The dynamic range in the abundance of these proteins could span a few orders of magnitude, wider than the range typically covered by a mass spectrometer [9]. For these reasons, comparative analyses, particularly (semi)quantitative analyses, of proteomic data of urine samples can be very challenging. This might be a key reason that there are no reliable urine markers for cancer diagnosis.

**Figure 1:**
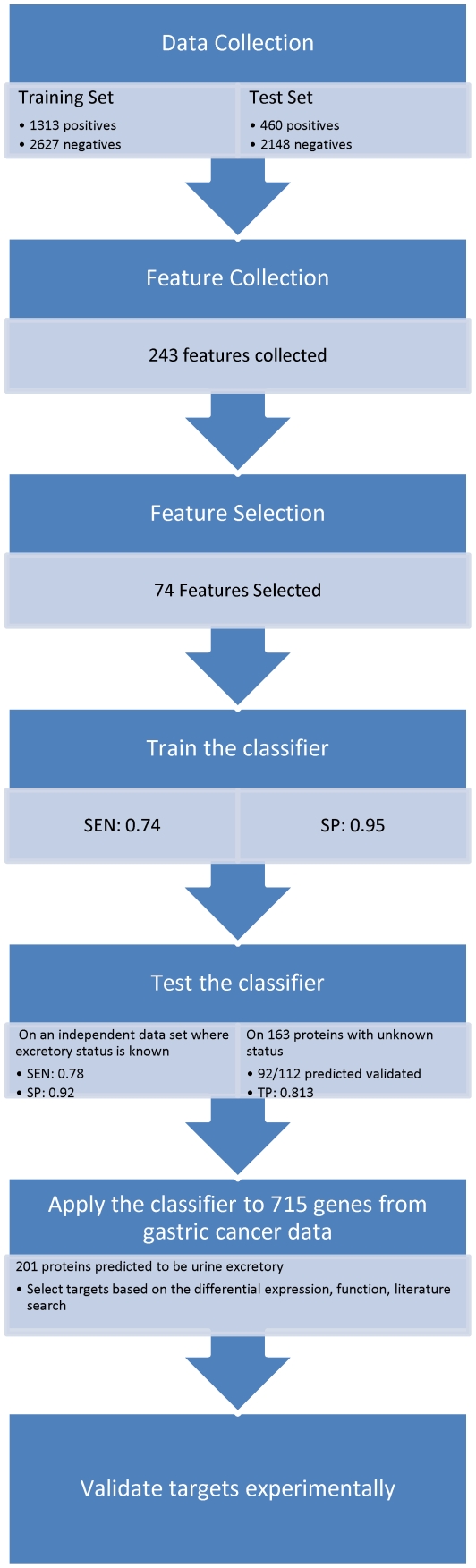
The flow of the study.

Our study focuses on development of a computational method for accurately predicting proteins that are urine excretory (see Figure 1 for the outline of the approach). These proteins must have specific properties that allow them to be secreted from cells first and then to be filtered out through the glomerulus membrane in kidneys. A recent proteomic study identified more than 1,500 proteins/peptides that are excreted into urine through healthy glomerular membranes [8]. Using this set of proteins and proteins deemed not to be urine excretory, we have identified a list of distinguishing features between these two classes of proteins and trained a support vector machine (SVM) based classifier to predict if a given protein might be excreted into urine. The prediction method was experimentally validated using antibody arrays in conjunction with Western blots, and the results are highly encouraging.

This classifier has been applied to predict proteins that might be excreted into urine based on the identified differentially expressed genes in gastric cancer *versus* reference gastric tissues; and a number of potential urine markers for gastric cancer have been identified. A key contribution made in this work is that it provides a new and effective way to guide proteomic studies of urine by suggesting candidate marker proteins, hence allowing targeted marker searches using antibody-mediated techniques like Western blots and Elisa, which are substantially more feasible than large-scale comparative proteomic analyses of urine samples without any targets with which to work. While this prediction program has been applied to gastric cancer data in this study, no gastric cancer-specific information was used in this program; hence, it can be used for urine marker searches for other diseases.

## Methods

This study consists of three main components: (i) construction of a classifier for predicting urine excretory proteins; (ii) evaluation of the performance of the classifier by applying it to a set of proteins for which the excretory status of the proteins is known; and (iii) application of the validated classifier to gene-expression data of gastric cancer to demonstrate its effectiveness in solving the urine marker identification problem.

This research was approved by the Institutional Review Board at the University of Georgia, Athens, Georgia, USA (Office of the Vice President for Research DHHS Assurance ID NO. FWA00003901, Project Number 2009-10705-1)and by the Chinese Institutional Review Board overseeing human subjects at Jilin University College of Medicine, Changchun, China. A consent form, approved by IRB at the University of Georgia and Chinese IRB, was collected from each subject. All subjects are aware that any data from research may be used for documents or publications as stated in the consent form.

### a. An algorithm for predicting excretory proteins

The general understanding of protein excretion from tissues to urine is that some proteins are secreted or leaked from cells into blood circulation, and then a portion of these proteins, along with some native proteins in blood, may be excreted into urine. Our goals are first to identify distinguishing features for such urine excretory proteins and then to build a classifier based on these features to predict which proteins in cells can be excreted into urine. To the best of our knowledge, there has not been any published work aimed to solve this problem. The importance in having such a capability is that it provides an effective link in connecting *omic* analyses of tissues to marker search in urine by providing candidate markers in urine that can be studied using antibody-based approaches.

The first step in developing such a predictive capability, i.e., a classifier, is to have a training dataset containing proteins that can and that cannot be excreted into urine, based on which a set of distinguishing features could possibly be identified. Fortunately, we have found one large proteomic dataset of urine samples from healthy people in a recently published study [8], which contains more than 1,500 unique proteins of which 1,313 have SwissProt accession IDs. We have used these 1,313 proteins as the positive training data for the to-be-trained classifier. The following procedure was then used to generate a negative training set: arbitrarily select at least one protein from each Pfam family that does not contain any positive training data, and the number of selected proteins from each family is proportional to the size of the family [10], [11]. As a result, 2,627 proteins were selected and used as the negative training set.

We examined 18 physiochemical features computed from protein sequences, which are potentially useful for the classification problem based on the general understanding of urinary excretion of proteins. The details of the 18 features and the computer programs used to calculate them are listed in Table S1. Some of these features are represented by multiple feature values, e.g., the amino acid composition in a protein sequence is represented by 20 feature values; overall the 18 features are represented using 243 feature values. We then identified a subset of features values from the 243, which can distinguish between the positive and the negative training data using an SVM-based classifier. The RBF kernel was used in our SVM training, considering its capability to handle non-linear attributes [12], [13].

To ascertain which of the initially considered features are actually useful, the feature selection tool provided in LIBSVM [12] was used to select the most discerning features among the 243. Other feature selection tools could possibly be used but we have considerable experience in using this tool and found it to be adequate. Codes used in this are publicly available from LIBSVM website (http://www.csie.ntu.edu.tw/~cjlin/libsvm/); we also have made the relevant program accessible at http://seulgi.myweb.uga.edu/files. An F-score [12], defined as follows, is used to measure the discerning power of each feature value to our classification problem,



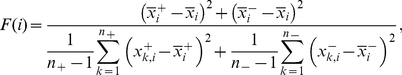
where 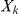 refers to the training feature values (k = 1,…, m); *n*_+_ and *n*_−_ are the number of proteins in the positive (+) and negative (-) training dataset, respectively; 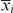, 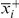, 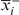 are the averages of the *i*th feature value across the whole training dataset, the positive dataset and the negative dataset, respectively; and 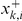 and 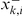 are the *i*th feature of the *k*th protein in the positive and negative training data, respectively. Generally, the larger an F-score, the more discriminative the corresponding feature is. In our selection, all features with F-scores above a pre-selected threshold were retained and used in training the final classifier. To find an optimal F-score threshold, we considered a list of possible thresholds and then selected the best one based on the training results.

The training of our SVM-based classifier is done using a standard procedure provided in LIBSVM [12] to find values of two parameters *C* and γ that give an optimal classification on the training data, where *C* controls the trade-off between training errors and classification margins, and γ determines the width of the kernel used [12]. Our training procedure is summarized as follows[12]:

- Obtain the F-score for each feature value;
- For each of the pre-selected thresholds, do the following
  - Remove the feature values with F-scores lower than the threshold;
  - Randomly split the training data into a sub-training and a sub-validation sets with equal size;
  - Train an SVM with an RBF kernel on the sub-training set to search for optimal values of *C* and γ, and then apply it to the sub-validation data and calculate the classification error;
  - Repeat steps (i) – (iii) five times and calculate the average validation error;
- Choose the threshold that gives the lowest average validation error, and keep the features with F-score above the selected threshold; and
- Retrain an SVM based on the selected features as the final classifier.

### b. Datasets used to evaluate the performance of the classifier

An independent dataset was used to assess the performance of the trained classifier for which the excretory status of each protein is known. The positive subset of this dataset has 460 human proteins found in the urine of healthy individuals by three urinary proteomics studies [14], [15], [16], and the negative subset contains 2,148 proteins selected using the same procedure described previously but does not overlap with the negative set used for training.

The following measures were used to assess the classification accuracies: the sensitivity, the specificity, the accuracy, the Matthew's correlation coefficient, and the AUC [17]. Table 1 summarizes the classification accuracies of the trained classifier on the both training and the test datasets [17]. From the classification accuracies on the two datasets, we believe that our trained classifier captured the key distinct features of the excretory proteins in urine.

**Table 1:** Classification performance by the trained classifier on the training and an independent test set.

Sets	TP	TN	FP	FN	SEN	SP	ACC	MCC	AUC
Train	972	2,493	134	341	0.74	0.95	0.88	0.52	0.94
Independent	360	1,983	165	100	0.78	0.92	0.90	0.45	0.93

TP = true positive; TN = true negative; FP = false positive; FN = false negative; N =  total number of proteins in dataset; SEN  =  TP/(TP+FN); SP  =  TN/(TN+FP); ACC  =  (TP+TN)/N; MCC  = (TPxTN-FPxFN)/√((TP+FN)(TP+FP)(TN+FP) (TN+FN)); AUC is described in (37).

In addition, our classifier was tested on a separate dataset, a subset of the 274 proteins fixed on a pre-made protein antibody array (the RayBio Human G-series Array 4000 (RayBiotech, Inc., Norcross, GA)). Of the 274 proteins, 111 are known to be excretory and were included in our training or independent test dataset. We applied the classifier on the remaining 163 proteins for which the excretory status was unknown (see Results and Table S2). This protein array provides the relative expression level for each protein on the array when tested on a (urine) sample, which is measured in terms of the signal intensity, quantified by the densitometry. The background of the array was used as the control to determine the actual presence of a protein in the (urine) sample. The signal intensity for a protein was considered as a true signal if it was at least 5-fold higher than that of the control, as suggested by the manufacturer's recommendation. We focused our experimental validation on confirming the positive predictions only since it is virtually impossible to prove a protein is not present in a urine sample due to limitations in detection sensitivity of the current technology when the protein is of very low concentration in the sample.

### c. Urine sample collection/preparation

Urine samples from gastric cancer patients and healthy controls were collected at the Medical School of Jilin University, Changchun, China. Gastric cancer patients, from who the samples were collected from, are all late stage patients (see Table S3 for patient information). These samples were immediately lyophilized and stored at −80°C until further use after their surgical removal from the patients. They were then reconstituted and centrifuged (3,000*xg* for 25 min at 4°C) to remove cellular components. The supernatants were collected and dialyzed at 4°C against Millipore ultra pure water (three buffer changes followed by an overnight dialysis) using Slide-A-Lyzer Dialysis Cassettes (Thermo Fisher Scientific, Rockford, IL). Protein concentrations were measured using the Bio-Rad Protein Assay (Bio-Rad, Hercules, CA) with bovine serum albumin as a standard.

### d. Identification of genes that are differentially expressed in gastric cancer and control tissues

A total of 80 gastric cancer tissues and their adjacent noncancerous tissues from 80 patients were collected at the Medical School of Jilin University. Microarray experiments were conducted on these tissues using the Affymetrix GeneChip Human Exon 1.0 ST Array, which covers 17,800 human genes. The PLIER algorithm [18] was used to summarize the probe signals to gene-level expressions. For each gene, we examined the distribution of the expression fold-change between the paired cancer and control tissues across all 80 pairs of tissues. Let *K_exp,_* be the number of pairs of tissues whose fold-change is at least 2. A gene is considered as *differentially expressed* if the *p*-value of the observed *K_exp_* is less than 0.05. Using this criterion, a total of 715 genes were found to be differentially expressed in gastric cancer across all human genes, and the names of the 715 genes, along with the associated *K_exp_* and *p*-values, are given in Table S4. A detailed study of the microarray data has been reported elsewhere [19].

### e. Function and pathway enrichment analyses

The DAVID Bioinformatics Resources and the KOBAS web server [20], [21] were used to do functional and pathway enrichment analysis, respectively, for all the predicted urine-excretory proteins, using the whole set of human proteins as the background. We refer the readers to [20], [21] for details on the methods for functional and pathway enrichment analyses. Using DAVID Bioinformatics Resources, the enrichment score for a specified group of proteins was determined by the EASE score [20], [22]. KOBAS is a complementary tool to DAVID as it expands the gene annotation using KEGG Orthology (KO) terms. The KOBAS web server, along with the KO-based annotation system [21], [23], was used to find statistically enriched and underrepresented pathways among the predicted urine-excreted proteins. KOBAS takes in a set of protein sequences and annotates them using the KO terms. The annotated KO terms were then compared against all human proteins as the background set for assessing if they are enriched or underrepresented.

### f. Western blots

Urinary proteins from each sample (total of 2 µg) were combined with 3x sample dye. Each tube was boiled for 5 min and loaded on SDS-PAGE gels, along with 10 µl standards and run for 1 h at 200 volts. The membrane was activated with 100% methanol, following a transfer from the gel to the membrane (100 volts for 1 h). Once the transfer was complete, the membrane was allowed to dry, rewetted in 100% methanol and washed 2X for 5 min each with Tris-Buffered Saline (TBS). The membrane was then incubated in 3% milk blocking solution for 2 h at room temperature. Next the membrane was incubated in the first antibody solution (1∶200 dilutions in 1.5% milk blocking) for 1 h at room temperature, and the unbound antibody was removed by washing the membrane 3X with TBS Tween-20 (TBST) solution for 10 min each. Then the membrane was incubated in a 1∶10,000 dilution of the secondary antibody in 1.5% milk blocking solution for 1 h at room temperature. The membrane was washed 3X with TBST and 2X with TBS (10 min each). Lastly, the membrane was covered completely with an equal amount of enhancer and peroxide solution from a Pierce Western Blotting kit for 5 min and exposed to the film. Each experiment was repeated multiple times to ensure reproducibility [24]. The signal intensities were determined using the imageJ software [25]. For each membrane, the blank lane was used to normalize the signal intensities across the membranes. The performance was examined using ROC and whisker-box plot.

## Results and Discussion

### a. Signal peptide and secondary structures are key features of urine-excreted proteins

The initial list of features was carefully selected to include what we believed to be protein characteristics relevant to urinary excretion based on literature search and our current understanding of urinary proteins. For example, the negatively charged glomerular wall in kidney will allow the filtration of only positively or neutrally charged proteins. Thus, charge of a protein is one of the features we selected. Taking the available information into consideration, the total number of feature values collected initially was 243, representing basic sequence properties, motifs, physicochemical properties, and structural properties (Table S1). In identifying features that are effective in discriminating urine excretory proteins from the non-excretory ones, a simple and effective method to eliminate features that show little or no discerning power for our classification problem was employed; 74 feature values were selected using the procedure outlined in Section a of Methods (Table S5). These feature values were used to train the final classifier.

Among the selected features, the most discriminatory one was the presence of signal peptides. It is understood that proteins that are secreted through the ER have signal peptides and are trafficked to their destination according to the specific signal peptides; thus, not surprisingly, most excreted proteins have this feature. Another prominent feature was the secondary structure type; specifically, the percentage of alpha helices in a protein sequence was ranked as the number 2 feature value among the selected 74 (Table S5). As expected, the charge of a protein was among the top ranked features for excreted proteins. This is consistent with the general understanding that charge is a factor in determining which proteins can be filtered through the glomerular membrane [26] as proteins inside glomerular membranes and podocyte slits are negatively charged, and hence negatively charged proteins will have low chances to filter through the kidneys. Indeed, the feature values of positive amino acids and charge were among the top ranked feature values.

Interestingly, however, molecular weight, which ranked at 232 out of 243, was not included in the final 74 feature values. This could be explained by the following. Proteins present in serum may have already undergone a cleavage or have been partially degraded, and thus may not be in their intact or complete form when they enter the kidney. It has, in fact, been established that the majority of proteins found in urine are extensively degraded [27]. While an intact protein may not be able to filter through the glomerulus due to its size or shape, a protein-derived peptide may easily pass through the podocyte slits. As a result, the molecular weight of the intact protein is a non-factor in predicting if the protein is urine excretory.

It should be noted that urine excretory proteins and secreted proteins share some common characteristics as some of the features used to identify blood-secreted proteins in our previous study [10] were selected in the urinary protein prediction in this study. For example, features such as solvent accessibility, polarity, and signal peptides were included in both classifiers. However there is a clear difference between the features used in the two classifiers. While features such as beta-strand-content, features associated with beta-barrel transmembrane protein and protein ratio, TatP motif, transmembrane domain, protein size, and the longest disordered region were among the top features for prediction of blood-secretory proteins [10], they were not included in the final features for the urinary protein prediction. Moreover, features related to positive charge, such as the composition of positively charged amino acids, were prominent in urinary protein prediction but not selected in the blood secretion prediction. Similarly, the alpha-helix-content and the coil-content of proteins were among the top features for urinary protein prediction, but they were not selected for the blood-secretory protein prediction. It is interesting to note that in contrast to the finding that beta-strands are a common secondary structure type among the blood secretory proteins, urinary proteins tend to have higher alpha-helix and coil content, which indicates that the urinary proteins possess properties not shared by blood secretory proteins in general.

### b. Performance of the classifier

To determine the accuracy of the final classifier, we tested it on an independent test set, which consists of 460 experimentally validated urine excretory proteins and 2,148 non-urine excretory proteins. Our classifier has its prediction sensitivity and specificity on this independent test set at 0.78 and 0.92, respectively (Table 1).

We then ran the classifier on the 163 out of the 274 proteins fixed on the pre-made antibody array (see Methods), for which the excretory status was unknown. Of the 163 proteins, 112 proteins were predicted to be urine excretory by our classifier. To assess the performance of this prediction, antibody array-based experiments were conducted on 14 urine samples, seven from healthy individuals and seven from gastric cancer patients. Of the 112 predicted urine-excretory proteins, 92 were found in at least one of the urine samples (Table S6), giving a positive prediction rate of 0.81, which is consistent with the performance level on the first test set.

It should be noted that one limitation of this classifier is that some proteins might have been partially degraded before being excreted into urine or in urine, making it difficult for our classifier to detect so formed peptides as it was trained on whole intact proteins. This issue will be addressed in the future through deriving feature values based on the actual proteins/peptides identified in previous urinary proteomic studies rather than their corresponding full-length proteins as done in this study. While there is clearly room for further improvement, the prediction results of the current classifier are highly encouraging.

### c. Application of classifier to gastric cancer data

Our previous study on 160 sets of microarray gene-expression data of gastric cancer has identified 715 differentially expressed genes with at least 2-fold changes in gastric cancer *versus* control tissue samples [19]. While it would be preferable to have proteomic data of the tissue samples, we have only gene-expression data available in this study. Hence, gene expression data are being used as an approximation to the protein expression in this methodology-oriented study. Our classifier was applied to these 715 proteins, and it predicted that 201 of the 715 proteins are urine excretory. Table S7 provides the detailed information of the 201 proteins. Since it is unrealistic to check all the 201 proteins in this study to determine if they are urine excretory or not, we did analyses to narrow down this list. Specifically, we have carried out the following analyses: (i) functional and pathway enrichment analyses to gain a better understanding of the types of proteins present in urine, (ii) literature search on urinary proteins to compile information about published urinary marker proteins, (iii) examining the gene expression data to remove genes that are not substantially differentially expressed between cancer and control tissue samples; and (iv) Western blots on proteins chosen from a narrowed down list of the 201 proteins. This procedure showed a high success rate and led to an interesting discovery of potential biomarker for gastric cancer.

For (i), we have carried out functional and pathway enrichment analyses on all the 201 proteins using the DAVID [20] and KOBAS [21] servers, respectively. We found that the enriched functional groups included the extracellular matrix (ECM), cell adhesion, and development, cell motility, defense response, angiogenesis, which are all known to be involved in the development of or in defense of cancer (Figure S1A). The most enriched pathways were ECM-receptor interaction and inorganic ion transport and metabolism pathways (Figure S1B).

The following criterion was used to reduce the list of 201 proteins for steps (ii) - (iii): *the proteins have not been reported to be related to any cancer based on our extensive literature search*, which gives rise to 71 proteins. The list was further reduced based on a pre-selected cutoff on differential expressions and functional annotations (potentially relevant to gastric cancer rather than immune responses).

### d. Endothelial lipase is substantially reduced in the urine samples of gastric cancer patients

We chose six proteins (MUC13, COL10A1, AZGP1, LIPF, MMP3, and EL) for experimental validation from the above narrowed down list. To do this, we have collected urine samples of 21 gastric cancer patients and 21 healthy individuals. Of the six selected proteins, five proteins, MUC13, COL10A1, LIPG, AZGP1, and EL were detected by Western blots in at least one urine sample. Out of the five, MUC13, COL10A1, and EL were detected even at a very low quantity of the total urinary proteins (1–2 µg). MMP3 was not found in the samples we tested, which may be due to the low concentration of MMP3 in urine or a false prediction by our classifier.

**Figure 2:**
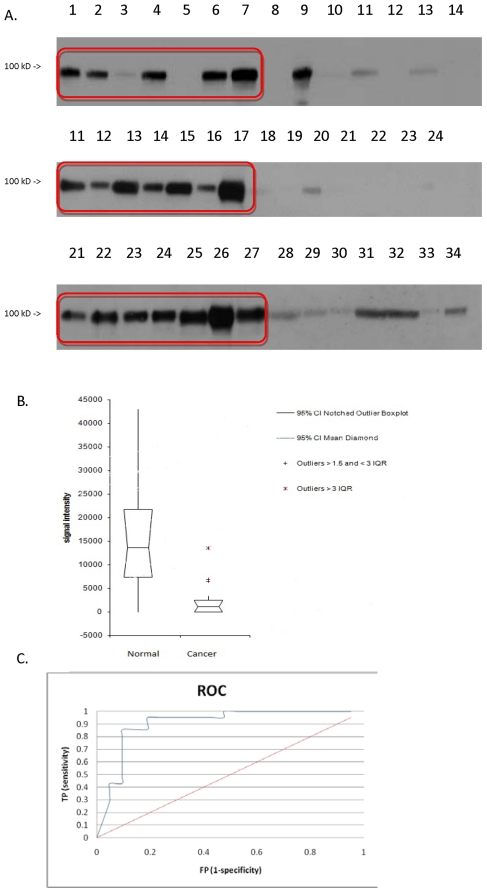
Western blot results. A: Western blots for EL on control and gastric cancer samples. Control samples (denoted by the red lined box): Lanes 1–7, 11–17, 21–27. Cancer samples: Lanes 8–14, 18–24, 28–34. B: Corresponding whisker-box plot for the signal intensities. C. ROC curve of the EL Western blot. Red line: no discrimination; blue line: ROC by EL.

It is particularly interesting to note that we were able to detect consistent differences in the EL abundance (encoded by *LIPG*) between the two sets of 21 urine samples. The Western blots for EL showed a substantial reduction in its abundance in urine samples of the 21 gastric cancer patients compared to the control samples. As shown in Figure 2A, the majority of the control samples showed the presence of EL, whereas most of the gastric cancer samples had relatively low amounts of EL. This pattern was observed repeatedly.

The molecular weight of this protein has been determined to be 68 kDa [28]; thus, a homo-dimer is expected to be 134 kDa. In the Western blots, however, bands were detected at near 100 kDa. This probably corresponds to a partially cleaved homo-dimer, an active form of which was confirmed by a previous study [29], although the possibility of a monomeric form of EL associated with another protein cannot be ruled out. The Western blots do provide semi-quantitative information based on the signal intensities. The ROC curve suggests that the EL concentration was discriminant in distinguishing the gastric cancer samples from the non-gastric cancer samples, yielding an AUC greater than 0.9 (Figure 2B–C). Using 5,000 as a signal intensity cutoff, true positive rate and false positive rate were 85% and 9.5%, respectively.

A further study is required to assess EL as a gastric cancer biomarker. The limited sample size of 21 samples in each group is too small to accurately evaluate its potential for biomarker. Enrolling many more patients is needed to confirm the efficacy of EL as a potential biomarker for clinical purposes. Also, it would be interesting to test EL on the early stage of gastric cancer, as our samples were all from late stage gastric cancer patients. Nonetheless, our preliminary result shows highly encouraging results.

### Concluding remarks

The available evidence indicates that many proteins are excreted into urine that may be good biomarker candidates for different diseases. The novel computational method developed and used herein for predicting excreted proteins may aid in identifying these and other biomarkers in urine. Our study has demonstrated that the integrated approach, coupling bioinformatics prediction with experimental validation, is an effective paradigm for identification and validation of potential urinary biomarkers. We anticipate that this approach will provide a powerful tool in the future for urinary proteomics and biomarker studies in general.

## Supporting Information

Figure S1 **Functional and pathway analyses.** A. Enriched functional groups as identified by DAVID. The x-axis represents the functional groups, and the y-axis represents the enrichment score. B. Enriched pathways for 201 predicted urine proteins using the KOBAS web server. Each blue bar represents the percentage of the 201 proteins; each red bar indicates all human proteins; the x-axis indicates the pathway names; and the y-axis indicates the percentage.
(TIFF)

Table S1 **Summary of features used in the initial classification model.** 1. Prilusky J, *et al.* (2005) FoldIndex: a simple tool to predict whether a given protein sequence is intrinsically unfolded. (Translated from eng) *Bioinformatics* 21(16):3435-3438 (in eng). 2. Li ZR, *et al.* (2006) PROFEAT: a web server for computing structural and physicochemical features of proteins and peptides from amino acid sequence. (Translated from eng) *Nucleic Acids Res* 34(Web Server issue):W32-37 (in eng). 3. Gasteiger E, *et al.* (2003) ExPASy: The proteomics server for in-depth protein knowledge and analysis. (Translated from eng) *Nucleic Acids Res* 31(13):3784-3788 (in eng). 4. Garrow AG, Agnew A, & Westhead DR (2005) TMB-Hunt: a web server to screen sequence sets for transmembrane beta-barrel proteins. (Translated from eng) *Nucleic Acids Res* 33(Web Server issue):W188-192 (in eng). 5. Bendtsen JD, Nielsen H, Widdick D, Palmer T, & Brunak S (2005) Prediction of twin-arginine signal peptides. (Translated from eng) *BMC Bioinformatics* 6:167 (in eng). 6. Kall L, Krogh A, & Sonnhammer EL (2007) Advantages of combined transmembrane topology and signal peptide prediction–the Phobius web server. (Translated from eng) *Nucleic Acids Res* 35(Web Server issue):W429-432 (in eng). 7. Julenius K, Molgaard A, Gupta R, & Brunak S (2005) Prediction, conservation analysis, and structural characterization of mammalian mucin-type O-glycosylation sites. (Translated from eng) *Glycobiology* 15(2):153-164 (in eng). 8. Gupta R, Jung E, & Brunak S (2004) Prediction of N-glycosylation sites in human proteins. 9. Eisenhaber F, Imperiale F, Argos P, & Froemmel C (1995) Prediction of Secondary Structural Content of Proteins from Their Amino Acid Comosition Alone Utilizing Analytic Vector Decomposition.
(DOC)

Table S2 **Uniprot IDs of 163 proteins used for classifier performance evaluation.**
(DOC)

Table S3 **Patient information for Western blot analyses.**
(DOC)

Table S4 **715 differentially expressed Genes in the gastric cancer tissues and the normal tissues.** The fold change K and the details of analysis are described in the paper http://csbl.bmb.uga.edu/~juancui/Publications/GC2009/Additional_material.pdf.
(XLS)

Table S5 **List of 74 features according to the rank.**
(DOC)

Table S6 **Experimental confirmation results of predicted urine excretory proteins (TP: true positive, FP: false positive).**
(DOC)

Table S7 **The list of 201 genes predicted to be excretory from differentially expressed genes of gastric cancer.**
(XLS)
