# Supplementary figures and images for: A Computational Method for Prediction of Excretory Proteins and Application to Identification of Gastric Cancer Markers in Urine
Source: PLoS One. 2011 Feb 18;6(2):e16875. doi: 10.1371/journal.pone.0016875 (PMC3041827; doi:10.1371/journal.pone.0016875)

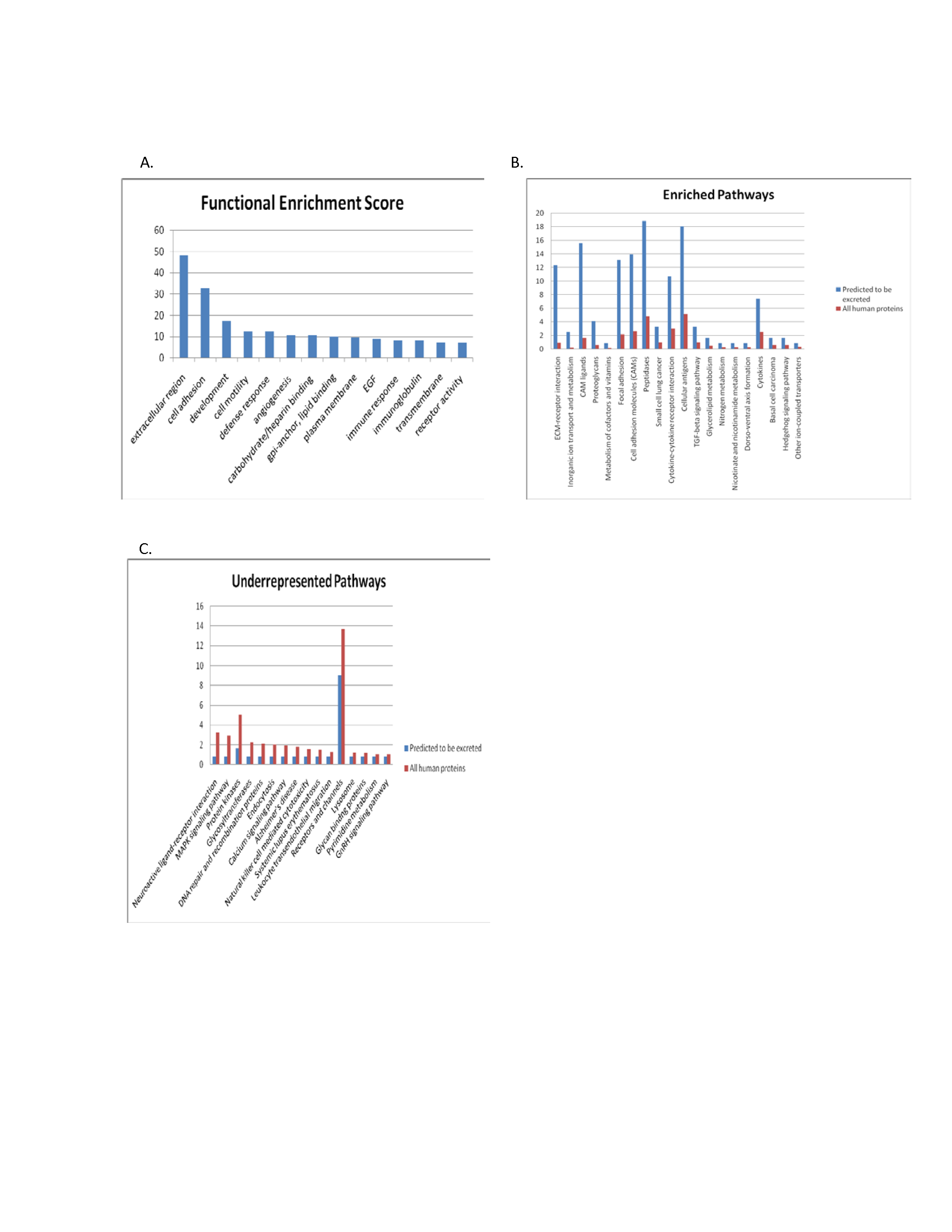

Supplement: Figure S1 — Functional and pathway analyses. A. Enriched functional groups as identified by DAVID. The x-axis represents the functional groups, and the y-axis represents the enrichment score. B. Enriched pathways for 201 predicted urine proteins using the KOBAS web server. Each blue bar represents the percentage of the 201 proteins; each red bar indicates all human proteins; the x-axis indicates the pathway names; and the y-axis indicates the percentage. (TIFF) [file pone.0016875.s001.tif]
