# Supplementary material for: A Computational Method for Prediction of Excretory Proteins and Application to Identification of Gastric Cancer Markers in Urine
Source: PLoS One. 2011 Feb 18;6(2):e16875. doi: 10.1371/journal.pone.0016875 (PMC3041827; doi:10.1371/journal.pone.0016875)
Supplement: Table S1 — Summary of features used in the initial classification model. 1. Prilusky J, et al. (2005) FoldIndex: a simple tool to predict whether a given protein sequence is intrinsically unfolded. (Translated from eng) Bioinformatics 21(16):3435-3438 (in eng). 2. Li ZR, et al. (2006) PROFEAT: a web server for computing structural and physicochemical features of proteins and peptides from amino acid sequence. (Translated from eng) Nucleic Acids Res 34(Web Server issue):W32-37 (in eng). 3. Gasteiger E, et al. (2003) ExPASy: The proteomics server for in-depth protein knowledge and analysis. (Translated from eng) Nucleic Acids Res 31(13):3784-3788 (in eng). 4. Garrow AG, Agnew A, & Westhead DR (2005) TMB-Hunt: a web server to screen sequence sets for transmembrane beta-barrel proteins. (Translated from eng) Nucleic Acids Res 33(Web Server issue):W188-192 (in eng). 5. Bendtsen JD, Nielsen H, Widdick D, Palmer T, & Brunak S (2005) Prediction of twin-arginine signal peptides. (Translated from eng) BMC Bioinformatics 6:167 (in eng). 6. Kall L, Krogh A, & Sonnhammer EL (2007) Advantages of combined transmembrane topology and signal peptide prediction–the Phobius web server. (Translated from eng) Nucleic Acids Res 35(Web Server issue):W429-432 (in eng). 7. Julenius K, Molgaard A, Gupta R, & Brunak S (2005) Prediction, conservation analysis, and structural characterization of mammalian mucin-type O-glycosylation sites. (Translated from eng) Glycobiology 15(2):153-164 (in eng). 8. Gupta R, Jung E, & Brunak S (2004) Prediction of N-glycosylation sites in human proteins. 9. Eisenhaber F, Imperiale F, Argos P, & Froemmel C (1995) Prediction of Secondary Structural Content of Proteins from Their Amino Acid Comosition Alone Utilizing Analytic Vector Decomposition. (DOC) [file pone.0016875.s002.doc]

| **Feature class** | **Features (No. of feature values** | **Program used to calculate the features** |
| --- | --- | --- |
| Sequence features | Sequence Length (1)  AA composition (20) | Fldbin (1), Profeat (2) |
| Physicochemical properties | Hydrophobicity (21), normalized Van der Waals volume (21), polarity (21), polarizability (21), charge (21), secondary structure (21), solvent accessibility (21), Pseudo-AA descriptor (50) | Locally calculated, Profeat (2): using three descriptors: composition, transition, and distribution |
|  | Unfoldability (1), charge (1), hydrophobicity (1), # of disordered regions (1), longest disordered regions (1), # of disordered residues (1), PI (1), MW (1), charge (2), percentage of disordered region (1) | Fldbin (1), Swiss-(3), locally calculated |
| Motifs | Transmembrane domain (1), Twin-arginine signal peptide (1), transemembrane domains (alpha helix, or beta barrel) (2), Glycosylation number & presence (N&O linked) (4) | TMB-Hunt (4-5), TatP (5), phobius (6), NetOgly (7), NetNGly (8) |
| Structural properties | Secondary structural content (4), Radius gyration (1), Radius (1), | SSCP (9), Radius Gyration (http://www.scfbio-iitd.res.in/software/proteomics/rg.jsp, locally calculated |
