# Supplementary material for: A Computational Method for Prediction of Excretory Proteins and Application to Identification of Gastric Cancer Markers in Urine
Source: PLoS One. 2011 Feb 18;6(2):e16875. doi: 10.1371/journal.pone.0016875 (PMC3041827; doi:10.1371/journal.pone.0016875)
Supplement: Table S2 — Uniprot IDs of 163 proteins used for classifier performance evaluation. (DOC) [file pone.0016875.s003.doc]

Table 2. List of 163 proteins used for classifier performance evaluation

| P03950 | P13232 | P09038 | P10145 | P22003 | P01135 | Q6EBC2 | Q15389 | P04271 |
| --- | --- | --- | --- | --- | --- | --- | --- | --- |
| O43927 | P13500 | P01138 | O14625 | P18075 | P61812 | P01308 | Q9BY76 | Q15465 |
| P12644 | P80075 | P35070 | P47992 | Q9H2A7 | P35590 | Q9BQR3 | Q15582 | P01266 |
| P22004 | P80098 | Q9NRJ3 | P10147 | O75509 | Q02763 | Q29983 | Q8WXI7 |  |
| P51671 | Q99616 | Q9Y4X3 | P13236 | P21860 | Q99727 | Q29980 | Q16790 |  |
| O00175 | O75078 | Q06418 | Q99731 | P16581 | P33151 | P22894 | P25774 |  |
| Q9Y258 | Q07325 | P42830 | P34130 | P48023 | P35968 | P09238 | Q16627 |  |
| P10767 | Q16663 | P25445 | O00300 | Q14627 | P35916 | P05121 | O00585 |  |
| P21781 | P78556 | P08620 | Q61207 | P31785 | P28908 | P02776 | P13385 |  |
| P80162 | P20783 | P31371 | P20333 | Q9HBE5 | P29965 | Q15109 | P41271 |  |
| P39905 | P55774 | P09919 | P19438 | Q01344 | P31994 | Q9Y6Q6 | P07585 |  |
| Q76BR7 | A2NWD3 | Q9UNG2 | O15444 | P15248 | Q8N4E7 | P02735 | O94907 |  |
| P08833 | Q92583 | Q9Y5U5 | P01033 | P02778 | O95633 | Q9Y336 | Q9UBP4 |  |
| P22692 | P01137 | P09341 | P40225 | P48357 | P19883 | P78536 | P27487 |  |
| P05019 | P10600 | O15467 | O14798 | P03956 | P09958 | Q96D42 | Q92838 |  |
| P22301 | P01375 | P14210 | Q9UBN6 | P45452 | P01241 | O14763 | P58294 |  |
| Q14005 | P01374 | P32942 | P15692 | P55773 | Q13651 | Q9NP99 | P04626 |  |
| P01583 | Q15848 | P08069 | O43915 | P16234 | Q9GZX6 | Q969D9 | P78552 |  |
| P60568 | O15123 | P29460 | Q13740 | P01236 | Q8IZJ0 | Q9HAV5 | Q9P0M4 |  |
| P05112 | P15514 | Q16552 | P33681 | O15389 | Q8IU54 | P02771 | Q96PD4 |  |
