# Supplementary material for: A Computational Method for Prediction of Excretory Proteins and Application to Identification of Gastric Cancer Markers in Urine
Source: PLoS One. 2011 Feb 18;6(2):e16875. doi: 10.1371/journal.pone.0016875 (PMC3041827; doi:10.1371/journal.pone.0016875)
Supplement: Table S3 — Patient information for Western blot analyses. (DOC) [file pone.0016875.s004.doc]

| **Lanes** | **Sample** | **Gender** | **Age Group** |
| --- | --- | --- | --- |
| 1 | N1 | M | 20-30 |
| 2 | N2 | M | 40-50 |
| 3 | N3 | M | 20-30 |
| 4 | N4 | M | 20-30 |
| 5 | N5 | M | 20-30 |
| 6 | N7 | M | 20-30 |
| 7 | N17 | F | 30-40 |
| 8 | C1 | F | 50-60 |
| 9 | C4 | F | 50-60 |
| 10 | C5 | M | 60-70 |
| 11 | C7 | F | 70-80 |
| 12 | C9 | M | 70-80 |
| 13 | C10 | M | 60-70 |
| 14 | C11 | M | 80-90 |
| 15 | N11 | F | 40-50 |
| 16 | N15 | F | unknown |
| 17 | N18 | M | unknown |
| 18 | N34 | M | 40-50 |
| 19 | N19 | M | unknown |
| 20 | N21 | M | 40-50 |
| 21 | N23 | F | 40-50 |
| 22 | C2 | F | 60-70 |
| 23 | C3 | M | 40-50 |
| 24 | C6 | M | 60-70 |
| 25 | C8 | M | unknown |
| 26 | C10 | M | 50-60 |
| 27 | C11 | M | 60-70 |
| 28 | C12 | F | 50-60 |
| 29 | N26 | M | 40-50 |
| 30 | N28 | M | 50-60 |
| 31 | N32 | M | 40-50 |
| 32 | N38 | M | 50-60 |
| 33 | N39 | F | 30-40 |
| 34 | N48 | M | 50-60 |
| 35 | N37 | M | 50-60 |
| 36 | C13 | F | 40-50 |
| 37 | C16 | unknown | unknown |
| 38 | C18 | M | 60-70 |
| 39 | C20 | M | 30-40 |
| 40 | C22 | M | 70-80- |
| 41 | C23 | M | unknown |
| 42 | C30 | F | 40-50- |
