# Supplementary material for: A Computational Method for Prediction of Excretory Proteins and Application to Identification of Gastric Cancer Markers in Urine
Source: PLoS One. 2011 Feb 18;6(2):e16875. doi: 10.1371/journal.pone.0016875 (PMC3041827; doi:10.1371/journal.pone.0016875)
Supplement: Table S5 — List of 74 features according to the rank. (DOC) [file pone.0016875.s006.doc]

| **Rank** | **Description** |
| --- | --- |
| 1 | presence of SP |
| 2 | Composition Secondary Structure: Helix (EALMQKRH) |
| 3 | Composition Normalized van der Waals vol. (range 0-2.78) |
| 4 | % of alpha-content |
| 5 | Transition Normalized van der Waals vol. (range 4.03-8.08) |
| 6 | Transition Secondary Structure: Coil (GNPSD) |
| 7 | Transition Polarizability value (.219-.409) KMHFRYW |
| 8 | Composition Charge. Positive (KR) |
| 9 | Composition Polarizability value (0-1.08) GASDT |
| 10 | Transition Polarizability value (0-1.08) GASDT |
| 11 | Composition Normalized van der Waals vol. (range 4.03-8.08) |
| 12 | Composition Polarizability value (.219-.409) KMHFRYW |
| 13 | % of coil-content |
| 14 | Amino acid composition G |
| 15 | Pseudo-AA descriptors |
| 16 | Amino acid composition T |
| 17 | Composition Secondary Structure: Coil (GNPSD) |
| 18 | Isoelectric point |
| 19 | Composition Charge. Neutral (ANCQGHILMFPSTWYV) |
| 20 | Transition Charge. Positive (KR) |
| 21 | Composition Hydrophobicity-neutral (GASTPHY) |
| 22 | Transition Normalized van der Waals vol. (range 0-2.78) |
| 23 | Transition Solvent Accessibility: Exposed(RKQEND) |
| 24 | Composition Polarity. Polarity Value(8.0-9.2) PATGS |
| 25 | Composition Polarity. Polarity Value(10.4-13.0) HQRKNED |
| 26 | Distribution |
| 27 | Pseudo-AA descriptors |
| 28 | Pseudo-AA descriptors |
| 29 | Distribution |
| 30 | Amino acid composition R |
| 31 | Composition secondary Structure: Strand (VIYCWFT) |
| 32 | Number of N-glyc site |
| 33 | Composition Hydrophobicity-polar (RKEDQN) |
| 34 | Composition Solvent Accessibility: Exposed(RKQEND) |
| 35 | Transition Polarity. Polarity Value(4.9-6.2) LIFWCMVY |
| 36 | Pseudo-AA descriptors |
| 37 | % of disordered region |
| 38 | Amino acid composition K |
| 39 | Amino acid composition C |
| 40 | Charge calculated |
| 41 | Distribution |
| 42 | Pseudo-AA descriptors |
| 43 | Pseudo-AA descriptors |
| 44 | Distribution |
| 45 | Amino acid composition M |
| 46 | Amino acid composition E |
| 47 | Pseudo-AA descriptors |
| 48 | Transition Charge. Neutral (ANCQGHILMFPSTWYV) |
| 49 | Distribution |
| 50 | Distribution |
| 51 | Transition Hydrophobicity-neutral (GASTPHY) |
| 52 | Transition Polarity. Polarity Value(8.0-9.2) PATGS |
| 53 | Composition Solvent Accessibility: Buried (ALFCGIVW) |
| 54 | Distribution |
| 55 | Pseudo-AA descriptors |
| 56 | Distribution |
| 57 | Composition Normalized van der Waals vol. (range 2.95-4.0) |
| 58 | Distribution |
| 59 | Transition Hydrophobicity-hydrophobic (CLVIMFW) |
| 60 | Charge |
| 61 | Pseudo-AA descriptors |
| 62 | Amino acid composition H |
| 63 | Unfoldability |
| 64 | Amino acid composition L |
| 65 | Distribution |
| 66 | Distribution |
| 67 | presence O-glyc site |
| 68 | Amino acid composition N |
| 69 | Distribution |
| 70 | Amino acid composition Y |
| 71 | Amino acid composition W |
| 72 | Pseudo-AA descriptors |
| 73 | Amino acid composition V |
| 74 | Pseudo-AA descriptors |
