# Supplementary material for: A Computational Method for Prediction of Excretory Proteins and Application to Identification of Gastric Cancer Markers in Urine
Source: PLoS One. 2011 Feb 18;6(2):e16875. doi: 10.1371/journal.pone.0016875 (PMC3041827; doi:10.1371/journal.pone.0016875)
Supplement: Table S6 — Experimental confirmation results of predicted urine excretory proteins (TP: true positive, FP: false positive). (DOC) [file pone.0016875.s007.doc]

| Protein ID | Experiment Result |
| --- | --- |
| P03950 | TP |
| P22004 | TP |
| P21781 | TP |
| P08833 | TP |
| P05019 | TP |
| P13500 | TP |
| O75078 | TP |
| P78556 | TP |
| P55774 | TP |
| P01137 | TP |
| P10600 | TP |
| Q15848 | TP |
| O15123 | TP |
| P15514 | TP |
| P01138 | TP |
| P35070 | TP |
| Q9NRJ3 | TP |
| Q06418 | TP |
| P42830 | TP |
| Q9UNG2 | TP |
| Q9Y5U5 | TP |
| P09341 | TP |
| O15467 | TP |
| P14210 | TP |
| P08069 | TP |
| P29460 | TP |
| Q16552 | TP |
| O14625 | TP |
| P47992 | TP |
| P10147 | TP |
| P13236 | TP |
| Q99731 | TP |
| P34130 | TP |
| P01033 | TP |
| P40225 | TP |
| O14798 | TP |
| O43915 | TP |
| Q13740 | TP |
| P22003 | TP |
| P18075 | TP |
| Q9H2A7 | TP |
| O75509 | TP |
| P21860 | TP |
| P16581 | TP |
| Q14627 | TP |
| P31785 | TP |
| Q01344 | TP |
| P15248 | TP |
| P02778 | TP |
| P48357 | TP |
| P45452 | TP |
| P55773 | TP |
| P16234 | TP |
| P01236 | TP |
| O15389 | TP |
| P01135 | TP |
| P61812 | TP |
| P35590 | TP |
| Q02763 | TP |
| P33151 | TP |
| P35968 | TP |
| P35916 | TP |
| P28908 | TP |
| Q8N4E7 | TP |
| O95633 | TP |
| P09958 | TP |
| P01241 | TP |
| Q8IZJ0 | TP |
| Q6EBC2 | TP |
| Q29983 | TP |
| Q29980 | TP |
| P22894 | TP |
| P05121 | TP |
| Q15109 | TP |
| Q9Y6Q6 | TP |
| Q9Y336 | TP |
| Q9NP99 | TP |
| P02771 | TP |
| Q15389 | TP |
| Q15582 | TP |
| Q16627 | TP |
| O00585 | TP |
| P07585 | TP |
| Q9UBP4 | TP |
| P27487 | TP |
| P04626 | TP |
| P78552 | TP |
| Q9P0M4 | TP |
| Q96PD4 | TP |
| Q15465 | TP |
| P01266 | TP |
| P12644 | FP |
| P80162 | FP |
| Q16663 | FP |
| P25445 | FP |
| P32942 | FP |
| O00300 | FP |
| Q61207 | FP |
| P20333 | FP |
| P19438 | FP |
| Q9HBE5 | FP |
| P03956 | FP |
| P19883 | FP |
| Q13651 | FP |
| P01308 | FP |
| Q9BQR3 | FP |
| P09238 | FP |
| P78536 | FP |
| O14763 | FP |
| Q16790 | FP |
| P25774 | FP |
| P41271 | FP |
